# Diagnostic utility of reticulocyte hemoglobin for iron-restricted anemia in patients with end-stage kidney disease on hemodialysis

**DOI:** 10.3389/fmed.2025.1689529

**Published:** 2025-11-06

**Authors:** Majed N. Almashjary, Ammar M. Sahl, Mohannad S. Hazzazi, Mohammad H. Alhashmi, Waleed M. Bawazir, Ammar A. Basabrain, Anwar Borai, Haitham Khalil, Mohammad Almohammadi, Malik A. Altayar, Abdullah M. Alqarni, Sahl A. Jamalallail, Salem M. Bahashwan, Husam Qanash, Elrashed B. Yasin

**Affiliations:** 1 Department of Medical Laboratory Sciences, Faculty of Applied Medical Sciences, King Abdulaziz University, Jeddah, Saudi Arabia; 2 Hematology Research Unit, King Fahd Medical Research Center, King Abdulaziz University, Jeddah, Saudi Arabia; 3 King Abdulaziz Medical City, Ministry of National Guard Health Affairs, Jeddah, Saudi Arabia; 4 Toxicology and Forensic Sciences Unit, King Fahd Medical Research Center, King Abdulaziz University, Jeddah, Saudi Arabia; 5 King Abdullah International Medical Research Center, King Saud Bin Abdulaziz University for Health Sciences, Pathology, King Abdulaziz Medical City, Jeddah, Saudi Arabia; 6 Department of Medical Laboratory Technology, Faculty of Applied Medical Sciences, University of Tabuk, Tabuk, Saudi Arabia; 7 Department of Hematology, Faculty of Medicine, King Abdulaziz University, Jeddah, Saudi Arabia; 8 Department of Medical Laboratory Science, College of Applied Medical Sciences, University of Ha'il, Hail, Saudi Arabia; 9 Medical and Diagnostic Research Center, University of Ha'il, Hail, Saudi Arabia; 10 Department of Medical Laboratory Technology, Faculty of Applied Medical Sciences, King Abdulaziz University, Rabigh, Saudi Arabia

**Keywords:** CKD, reticulocyte hemoglobin, IDA, anemia, ACD, hemodialysis

## Abstract

**Background:**

Chronic kidney disease (CKD) is a serious, long-term illness that damages kidneys and lowers glomerular filtration rate. CKD often causes anemia. Iron deficiency (ID) is common in these patients and worsens illness symptoms. Modern hematology analysers can measure reticulocyte mean cell hemoglobin (MCHr), which directly measures iron integration into erythrocyte hemoglobin. MCHr can improve iron deficiency detection in CKD patients, who have aberrant iron indicators due to chronic inflammation. This research aims to evaluate the effectiveness of MCHr as a marker for ID in patients with CKD.

**Method:**

To obtain data for this study, CBC, reticulocyte profile, and iron biomarkers were collected from King Khalid National Guard Hospital (Ref No. IRB/1861/23). Transferring saturation was calculated using (Serum iron/TIBC) × 100. GraphPad Prism 9 software was used to analyze the data, and Mann-Whitney, Spearman correlation, and ROC plots were used to determine MCHr's diagnostic performance. A *p*-*value* < 0.05 was considered significant.

**Result:**

The study compared 190 individuals undergoing hemodialysis (HD) with 165 healthy blood donors. The HD group showed lower levels of RBC, Hb, MCHr, serum iron, and total iron-binding capacity (TIBC). Despite anemia, the HD group had higher levels of ferritin and transferrin saturation (TSAT). MCHr demonstrated excellent diagnostic performance in identifying iron deficiency anemia (IDA), particularly in functional iron deficiency. When TSAT was < 20%, MCHr showed an AUC of 0.98, with 100% specificity and 72.41% sensitivity, significantly outperforming ferritin and TSAT in inflammatory settings. In the HD group with ferritin levels < 200 ng/mL, the MCHr cut-off value of < 31.20 pg had a sensitivity and specificity of 89.47% and an AUC of 0.89. When TSAT was < 20%, the MCHr cut-off value of < 23.95 pg had a sensitivity of 72.41%, specificity of 100%, and AUC of 0.98.

**Conclusions:**

Based on the findings, MCHr is more effective than ferritin and TSAT in detecting iron deficiency in hemodialysis patients. Future research should use MCH to investigate the impact of iron therapy with or without rHuEPO.

## 1 Introduction

Chronic kidney disease (CKD) represents a growing global health burden, affecting more than 10% of the world's population and contributing significantly to cardiovascular morbidity, reduced quality of life, and increased mortality (1, 2). The KDIGO guidelines define chronic kidney disease as abnormalities of kidney structure or function that are present for more than 3 months and have health implications (3). However, it is important to note that progression to end-stage kidney disease (ESKD) can be rapid in certain conditions, such as rapidly progressive glomerulonephritis (RPGN) or systemic vasculitis, and does not always follow a prolonged course. These accelerated scenarios are clinically relevant, especially when assessing complications like anemia. In Saudi Arabia, CKD is among the top 10 causes of death, underscoring its public health impact and the necessity for timely diagnosis and comprehensive management strategies (4).

One of the clinically important complications of CKD is anemia, which becomes more prevalent and severe with progressive loss of renal function, particularly in patients receiving hemodialysis (5, 6). The causes of anemia in CKD are multifactorial, including reduced erythropoietin (EPO) production, shortened red blood cell (RBC) lifespan, chronic inflammation, and disordered iron homeostasis (7). Among these, iron deficiency whether absolute or functional is a key contributor to anemia, limiting the effectiveness of erythropoiesis-stimulating agents (ESAs) and worsening patient outcomes. In addition to poor dietary intake or overt blood loss, iron deficiency in CKD may also result from functional mechanisms such as inflammation-driven hepcidin elevation, volume overload, intestinal mucosal edema, impaired absorption due to phosphate binders, polypharmacy, and complications of secondary hyperparathyroidism (SHPT), all of which impair iron mobilization and utilization (8). These pathophysiologic mechanisms—especially reduced erythropoietin synthesis and iron deficiency represent the two principal contributors to anemia in CKD, and form the clinical foundation for our study's diagnostic focus.

Traditionally, the diagnosis of iron deficiency in CKD has relied on serum ferritin and transferrin saturation (TSAT). However, these markers are limited by their acute-phase reactivity, making them unreliable in the setting of inflammation, which is common in hemodialysis patients (9). Ferritin levels may be elevated despite iron depletion, and TSAT levels can fluctuate considerably due to inflammation or recent iron administration, leading to potential under- or overtreatment (10).

In response to these limitations, recent guidelines and studies have advocated for the use of more functional markers of iron availability, particularly reticulocyte hemoglobin content (CHr or MCHr) (11). MCHr reflects the hemoglobin content in newly produced reticulocytes and serves as a dynamic measure of iron bioavailability for erythropoiesis over the previous 2–3 days (12, 13). This real-time characteristic makes MCHr particularly suitable for identifying iron-restricted erythropoiesis, including in the setting of functional iron deficiency, where total body iron is adequate but not effectively utilized (14).

Several studies have supported the clinical utility of MCHr in diagnosing iron deficiency across diverse patient populations. Miwa et al. demonstrated its value in HD patients, showing rapid response to intravenous iron and strong correlation with established markers (15). Almashjary et al. (17) recently validated MCHr cut-offs for detecting iron deficiency and anemia in Saudi populations, highlighting its applicability in Middle Eastern cohorts. In addition, the Kidney Disease: improving Global Outcomes (KDIGO) guidelines now recognize the potential role of reticulocyte indices, including CHr, in optimizing anemia management in CKD (3).

In this study, we aim to evaluate the diagnostic accuracy of MCHr in detecting iron-restricted erythropoiesis among Saudi patients with end-stage kidney diseases (ESKD) undergoing hemodialysis. By comparing MCHr against traditional markers like ferritin and TSAT and establishing optimal cut-off values, this research seeks to support the integration of MCHr into routine clinical protocols for more accurate, timely, and individualized anemia management in CKD.

## 2 Materials and methods

### 2.1 Study design and participants

This cross-sectional case-control study involved 190 CKD patients classified as ESKD undergoing hemodialysis and 165 healthy donors as controls. Inclusion criteria encompassed adults aged 18 years or older with ESKD undergoing regular hemodialysis for at least 3 months. Patients with recent blood transfusions (within the last 3 months), active infections, or malignancies were excluded. Ethical approval was obtained from King Abdullah International Medical Research Center (Ref No. IRB/1861/23).

### 2.2 Sample collection and biochemical analyses

Blood samples were collected under standardized conditions using EDTA tubes for CBC and reticulocyte analysis and serum separator tubes for iron and inflammation biomarkers. CBC and reticulocyte indices were measured using the Alinity hq, a modern automated hematology analyzer (Abbott Laboratories, IL, USA) that uses the Multiangle Polarized Scatter Separation (MAPSS) principle. Serum ferritin, iron, total iron-binding capacity (TIBC), and C-reactive protein (CRP) were quantified using the Architect system c8000 and i2000 (Abbott Laboratories, IL, USA) chemiluminescence and colorimetric assays.

TSAT was calculated as (serum iron/TIBC) × 100. Anemia was defined as hemoglobin < 13 g/dL for men and < 12 g/dL for women, per World Health Organization criteria. Iron deficiency was operationally defined based on KDIGO guidelines and supported by prior literature as ferritin < 100 ng/mL and/or TSAT < 20%. For severe IDA subgroup analysis, ferritin thresholds of < 200 ng/mL and < 500 ng/mL were used to stratify inflammatory bias in ferritin interpretation. These definitions are consistent with clinical diagnostic standards in hemodialysis populations (18).

### 2.3 Statistical analysis

Group differences were assessed using Mann-Whitney U tests for continuous variables. Spearman's Correlation coefficients evaluated the relationships between MCHr and other iron biomarkers. Receiver operating characteristic (ROC) curves were constructed to determine MCHr's diagnostic efficacy, calculating sensitivity, specificity, and area under the curve (AUC). In addition, to address potential demographic confounding, we performed an analysis of covariance (ANCOVA) with MCHr as the dependent variable, group (ESKD vs. control) as the fixed factor, and age as a covariate. This allowed us to assess whether differences in MCHr between groups remained significant after adjusting for age. For a subset of patients with available CRP data (*n* = 94), exploratory analyses were performed to assess the association between inflammation and MCHr. Pearson and Spearman correlation coefficients were calculated between CRP and MCHr values. This allowed us to evaluate the potential influence of inflammation on MCHr compared with conventional iron markers. Statistical significance was set at *p* < 0.05, and all analyses were performed using GraphPad Prism 9 (GraphPad Software, San Diego, CA, USA).

## 3 Results

### 3.1 Participants' demographics

A total of 355 participants were included in the study: 190 ESKD patients on hemodialysis and 165 healthy donors as controls. There were 104 (54.7 %) males and 86 (45.3 %) females as ESRF patients, 139 (84.2 %) males, and 26 (15.8 %) females as healthy donors. The average age of ESRF patients was 59 ± 15.6, while the average age of healthy donors was 29 ± 10.5 years (Table 1).

**Table 1:** Participants' demographic and clinical information.

**Variables**	**Age and gender**	**ESKD (*n* = 190) *n* (%)**	**Control (*n* = 165) *n* (%)**
Age	18–29	8 (4.2)	121 (73.3)
	30–49	42 (22.1)	34 (20.6)
	>50	140 (73.1)	10 (6.1)
Gender	Male	104 (54.7)	139 (84.2)
	Female	86 (45.3)	26 (15.8)

### 3.2 Participants' clinical findings

The ESKD patients group had significantly lower in hematological parameters: RBC, Hb, MCHr, and in iron biomarkers: serum iron and TIBC when compared to the control group (RBC: 3.90 ± 0.65 vs. 4.95 ± 0.45: *p* < 0.0001, Hb: 11.6 ± 1.46 vs. 14.3 ± 1.18: *p* < 0.0001, MCHr: 29.9 ± 4.1 vs. 33.2 ± 2.59; *p* < 0.0001, serum iron: 11.1 ± 4.68 vs. 15.8 ± 5.63: *p* < 0.0001, TIBC: 34.92 ± 7.02 vs. 54.17 ± 6.49: *p* < 0.0001) (Table 2). Despite the anemia, MCV, MCH, ferritin, and TSAT were significantly higher in the ESKD group (MCV: 93.3 ± 8.1 vs. 88.69 ± 5.65; *p* < 0.0001, MCH: 30.1 ± 2.6 vs. 28.93 ± 1.82; *p* < 0.0001, ferritin: 596.6 ± 356.1 vs. 92.38 ± 64.37; *p* < 0.0001, TSAT: 32.16 ± 12.62 vs. 29.44 ± 10.95, *p* = 0.001) (Table 2). To further account for the age imbalance between groups, we conducted an age-adjusted analysis of covariance (ANCOVA). The difference in MCHr between dialysis patients and healthy controls remained statistically significant after adjusting for age (β = −2.99 pg, 95% CI: −4.09 to −1.90, *p* < 0.0001). These findings confirm that the lower MCHr observed in the ESKD group is independent of age.

**Table 2:** Participants' clinical findings.

**Parameters**		**Control (mean ±SD)**	**ESKD (mean ±SD)**	***p-value***
Hematological parameters	RBC (106/uL)	4.95 ± 0.45	3.90 ± 0.65	< 0.0001
	Hb (g/dL)	14.3 ± 1.18	11.6 ± 1.46	< 0.0001
	MCV (fL)	88.69 ± 5.65	93.3 ± 8.1	< 0.0001
	MCH (pg)	28.93 ± 1.82	30.1 ± 2.6	< 0.0001
	MCHC (g/dL)	32.64 ± 0.97	32.27 ± 0.75	0.0003
	MCHr (pg)	33.2 ± 2.59	29.9 ± 4.1	< 0.0001
Iron parameters	Ferritin (ng/mL)	92.38 ± 64.37	596.6 ± 356.1	< 0.0001
	Serum Iron (umol/L)	15.8 ± 5.63	11.1 ± 4.68	< 0.0001
	TSAT (%)	29.44 ± 10.95	32.16 ± 12.62	0.0230
	TIBC (umol/L)	54.17 ± 6.49	34.92 ± 7.02	< 0.0001

### 3.3 The association between MCHr with iron and inflammation biomarkers

**Figure 1:**
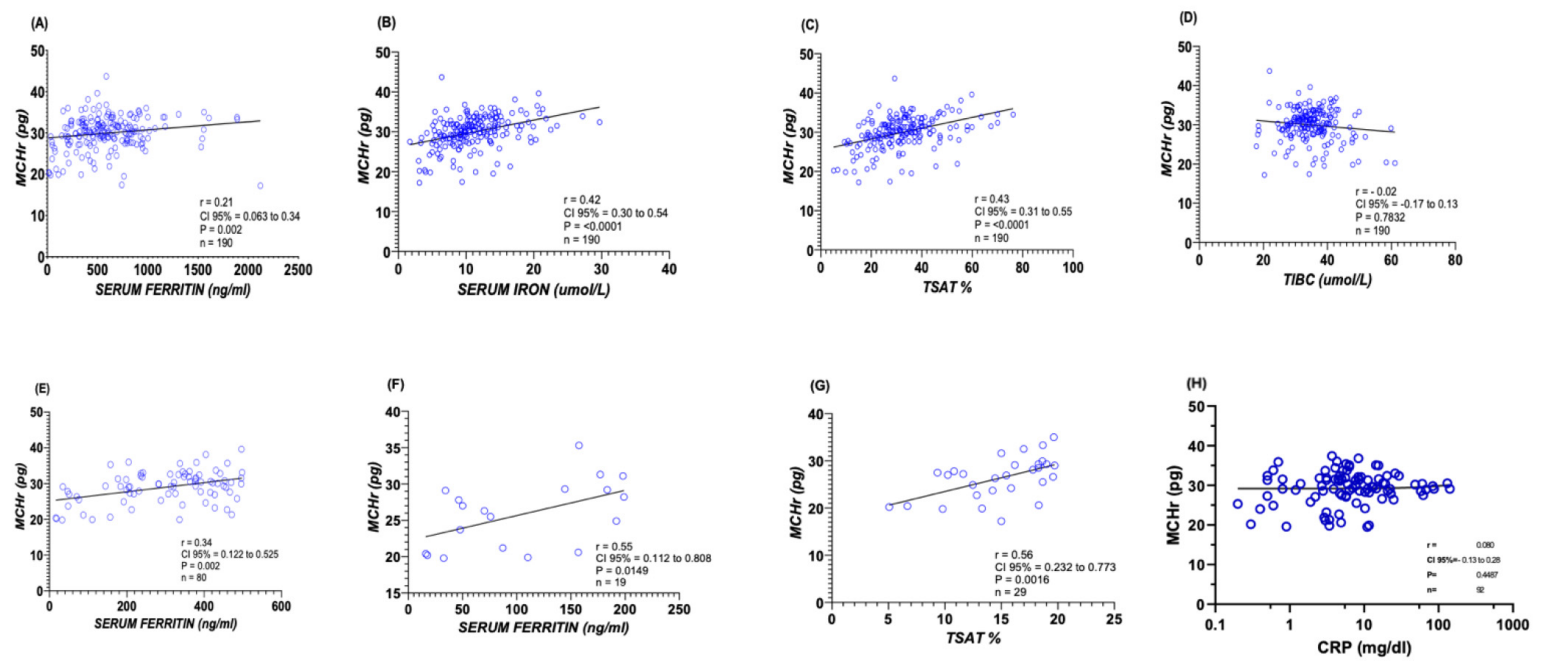
Correlations between MCHr and iron-related biomarkers in ESKD patients. **(A)** Ferritin (*n* =190), **(B)** Serum iron (*n* = 190), **(C)** Transferrin saturation (TSAT) (*n* =190), **(D)** TIBC (*n* = 190), **(E)** Ferritin < 500 ng/mL (*n* = 80), **(F)** Ferritin < 200 ng/mL (*n* = 19), **(G)** TSAT < 20% (*n* =29), and **(H)** CRP (*n* = 94). Spearman's correlation coefficient (r) and *p-values* are shown—statistical test: Spearman's rank correlation.

Spearman's rank correlation analysis was employed to assess the relationship between MCHr and various iron biomarkers rigorously. The analysis revealed a statistically significant positive correlation between MCHr and ferritin, with a correlation coefficient (r) of 0.21 and a *p-value* of 0.002 (Figure 1A). Further, a moderate positive association was observed between MCHr and serum iron (*r* = 0.42, *p* < 0.0001; Figure 1B), as well as with TSAT (*r* = 0.43, *p* < 0.0001; Figure 1C). Conversely, non-significant negative correlation was noted between MCHr and TIBC (*r* = −0.02, *p* = 0.7832, Figure 1D).

In a more focused analysis of severe IDA cases in ESRF patients, where ferritin levels were below 500 ng/ml and 200 ng/ml, a statistically positive correlation was observed with MCHr when ferritin level below 500 ng/mL (*r* = 0.34, *p* = 0.002; Figure 1E), and show a significant positive correlation with MCHr when ferritin level were below 200 ng/mL (*r* = 0.55, *p* = 0.0149; Figure 1F). Similarly, when TSAT percentages were under 20, a positive correlation with MCHr was evident (*r* = 0.56, *p* = 0.0016; Figure 1G). In a subset of 94 ESKD patients with available CRP measurements, MCHr demonstrated only a very weak and non-significant correlation with CRP (Spearman *r* = 0.08). These findings suggest that MCHr is minimally affected by inflammation, consistent with its proposed stability compared with ferritin and TSAT in inflamed patients (Figure 1H).

### 3.4 The utility of MCHr as a diagnostic marker for IDA

**Figure 2:**
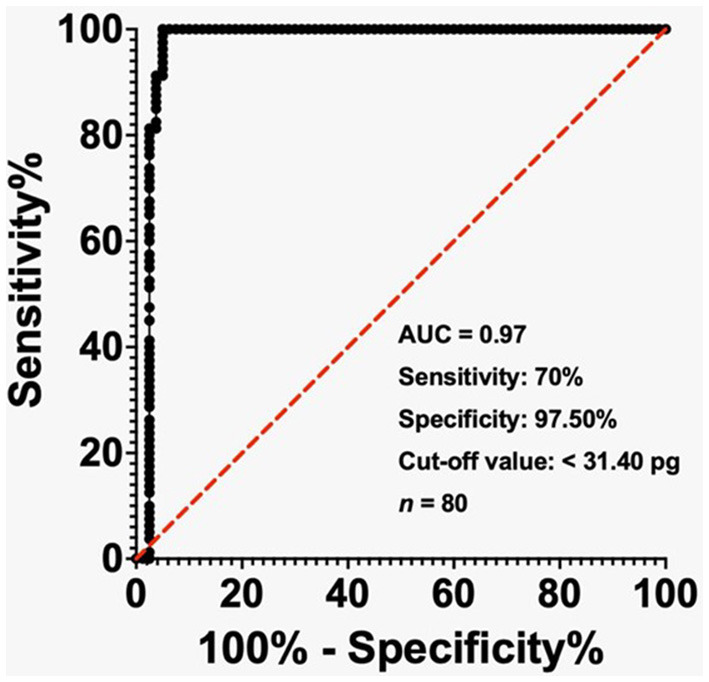
Receiver-operating characteristic (ROC) curve showing the diagnostic performance of MCHr for iron deficiency anemia (IDA) in ESKD patients with ferritin < 500 ng/mL. The cut-off value of 31.40 pg yielded 70% sensitivity and 97.5% specificity. Area under the curve (AUC) = 0.97. Sample size: 80 ESKD.

**Figure 3:**
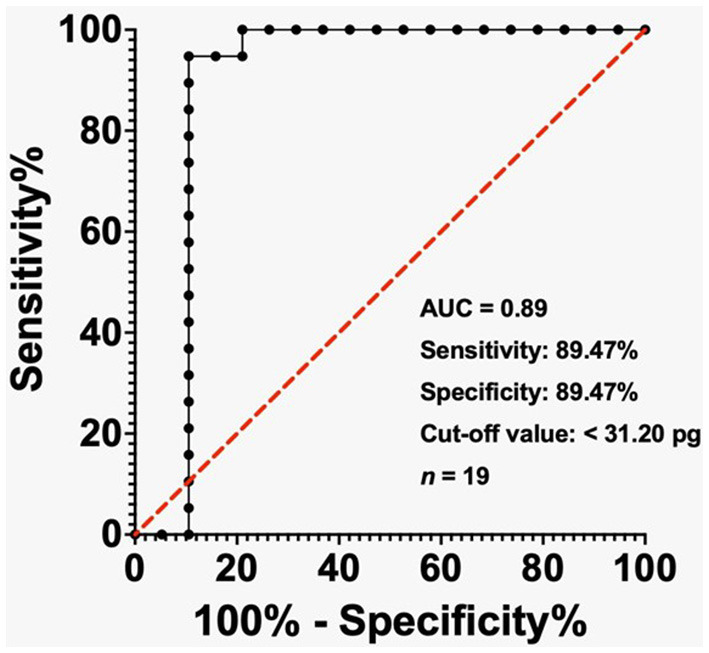
ROC analysis of MCHr in diagnosing IDA in ESKD patients with ferritin < 200 ng/mL. The cut-off value of 31.20 pg yielded 89.47% sensitivity and 89.47% specificity. AUC = 0.89. Sample size: 19 ESKD patients. Statistical test: ROC analysis.

**Figure 4:**
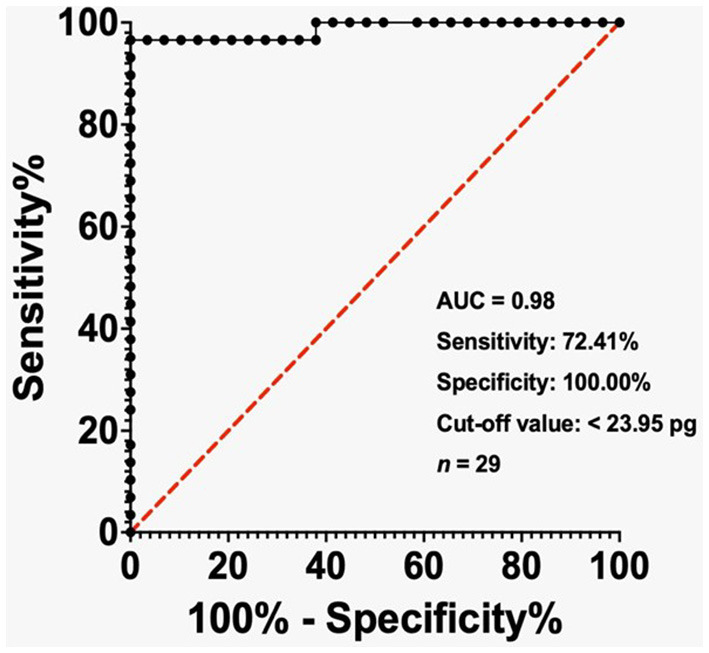
ROC analysis of MCHr in diagnosing IDA in ESKD patients with TSAT < 20%. The cut-off value of 23.95 pg yielded 72.41% sensitivity and 100% specificity. AUC = 0.98. Sample size: 29 ESKD patients. Statistical test: ROC analysis.

The ROC visually displays how well a binary classification model performs. It illustrates the relationship between the true and false positive rates at various classification thresholds. The AUC represents the area under the ROC curve. The diagnostic potential of MCHr as a marker for IDA when serum ferritin level < 500 ng/mL, < 200 ng/mL, and TSAT < 20% was evaluated by conducting ROC study. The results were then graphically represented in Figures 2–4.

In the ESKD group, the AUC reached 0.97 when the ferritin level was below 500 ng/mL, while the cut-off value of less than 31.40 pg exhibited a sensitivity of 70% and a specificity of 97.50% (Figure 2). The AUC was 0.89 in the ESKD group when the ferritin level was < 200 ng/mL, and the cut-off value of < 31.20 pg had sensitivity and specificity of 89.47% (Figure 3). Finally, in the ESKD group where TSAT was less than 20%, the AUC reached 0.98. Additionally, the cut-off value below 23.95 pg yielded a sensitivity of 72.41% and a specificity of 100% (Figure 4).

## 4 Discussion

Anemia in CKD, particularly in ESKD patients undergoing hemodialysis, is multifactorial in origin and poses substantial challenges to clinical management. Inflammation-driven functional iron deficiency (FID), inadequate EPO production, and disrupted iron homeostasis are central mechanisms that contribute to impaired erythropoiesis in this population (7, 8, 19). Importantly, the inflammatory milieu of ESKD leads to increased hepcidin production, which inhibits the iron exporter ferroportin and prevents iron release from macrophages and intestinal cells, resulting in poor iron availability despite sufficient stores (20).

This study highlights the diagnostic superiority of MCHr in detecting iron-restricted erythropoiesis among ESKD patients. MCHr reflects the hemoglobinization of young red blood cells over the previous 2–3 days, providing a real-time picture of iron supply to the bone marrow. In contrast to conventional markers such as serum ferritin and TSAT, which are heavily influenced by inflammation, MCHr appears to be mainly unaffected by acute-phase reactions and better correlates with iron bioavailability (12, 13, 16).

Our findings agree with earlier reports demonstrating the diagnostic and prognostic value of MCHr and its equivalent parameters CHr (Siemens) and Ret-He (Sysmex) in both dialysis and non-dialysis populations. Miwa et al. reported that MCHr was a reliable indicator of iron availability in HD patients and could respond rapidly to iron therapy, showing changes within 2 weeks of intravenous supplementation (15). Similarly, studies by Brugnara et al. (12) and Thomas et al. (13) emphasized MCHr's value in detecting early stages of functional iron deficiency, which are frequently missed by standard iron indices.

Our ROC analysis revealed high diagnostic performance for MCHr in both absolute and functional iron deficiency. At a TSAT threshold < 20%, an MCHr cut-off of < 23.95 pg provided 100% specificity and an AUC of 0.98—outperforming ferritin and TSAT, which can be elevated or falsely normal in inflammatory states (21, 22). Our findings show that MCHr achieved an AUC of 0.98 with 100% specificity when TSAT < 20%, significantly outperforming conventional markers whose diagnostic accuracy is confounded by inflammation. These results echo previous findings by Miwa et al. (15) and Sany et al. (7) and provide new, population-specific validation of MCHr's diagnostic reliability in a Middle Eastern cohort (16).

Additionally, our study found strong correlations between MCHr and serum iron (*r* = 0.62), TSAT (*r* = 0.55), and TIBC, while the correlation with ferritin was weak and inverse in patients with inflammation. This reinforces the concept that MCHr more accurately reflects marrow iron supply and should be prioritized when diagnosing iron deficiency in ESKD on hemodialysis settings, particularly in patients with elevated inflammatory markers (10, 20).

The use of MCHr as a therapeutic guide has also gained traction. Fishbane et al. (14) described MCHr as a potential monitoring tool that can identify patients likely to benefit from iron or ESAs, thereby helping tailor treatment. Furthermore, KDIGO guidelines now acknowledge the role of reticulocyte indices like CHr and Ret-He as supportive tools in assessing iron status and therapy response (3).

In our study, MCHr values were consistently lower in ESKD patients than in healthy controls, despite elevated ferritin levels. This aligns with the findings of Szczech et al. (23), who observed that high ferritin may not indicate adequate iron availability in dialysis patients, particularly in the presence of inflammation or malnutrition. The clinical implication is profound: reliance on static markers may delay necessary iron therapy or expose patients to iron overload risks.

Another important observation is the potential for MCHr to predict ESA responsiveness. Low MCHr levels have been associated with hyporesponsiveness to EPO therapy and increased mortality in dialysis patients, underscoring its prognostic relevance (24, 25). Prospective studies have shown that correcting MCHr levels improves hemoglobin response and reduces ESA doses, making it a target for anemia management algorithms (26, 27).

A notable demographic limitation of this study is the age difference between the ESKD and control groups (mean age: 59 vs. 29 years). Age-related hematological variation, including reduced marrow responsiveness and altered RBC indices, could theoretically influence baseline MCHr values. However, previous studies have shown minimal age-related effects on MCHr in adults, and its diagnostic performance is expected to remain robust because it primarily reflects current iron availability rather than cumulative marrow health (13). Our ANCOVA analysis further confirmed that the observed differences in MCHr remained significant after adjusting for age, although residual confounding cannot be entirely excluded. Future studies with age- and sex-matched controls would provide stronger validation.

In addition, CRP data were available for a subset of 94 patients, and exploratory analyses demonstrated no meaningful correlation between CRP and MCHr (Pearson *r* = 0.04; Spearman *r* = 0.08). This supports the relative independence of MCHr from inflammatory effects, whereas ferritin is well established to correlate strongly with CRP and other acute-phase reactants. Nevertheless, larger studies incorporating broader inflammatory profiling, including biomarkers such as IL-6, are warranted to confirm these findings.

Another limitation is that detailed information on patients' comorbidities and medication use was not available. These factors, such as chronic illnesses or treatments like erythropoiesis-stimulating agents and iron therapy, may influence MCHr values and should be included in future studies to refine its diagnostic accuracy. Because detailed information on these variables was unavailable, statistical adjustment for potential confounders could not be performed. Nevertheless, the strong discriminatory performance of MCHr between ESKD patients and controls, along with its biological stability compared with ferritin and TSAT, suggests that these factors are unlikely to have materially biased the findings. Future work should incorporate comprehensive clinical and pharmacologic data to enable formal multivariable adjustment.

Finally, although MCHr is routinely available on automated hematology analyzers, a lack of standardization in terminology and reference ranges across platforms (CHr, Ret-He, MCHr) remains a challenge for clinical interpretation. As highlighted by Brugnara and colleagues, harmonization of nomenclature and thresholds is essential for wider adoption. While CHr (Siemens), Ret-He (Sysmex), and MCHr (Abbott) all measure reticulocyte hemoglobin content, inter-assay variability persists. Comparative calibration studies and universal reference intervals are needed to achieve cross-platform harmonization and ensure consistency in clinical practice (12). Future validation should include multi-center studies directly comparing MCHr across platforms, establishing conversion algorithms or analyzer-specific cut-offs, and working toward the development of unified international reference standards. Such harmonization efforts would ensure that the diagnostic utility of MCHr can be applied broadly across different laboratory settings.

Unlike prior studies conducted primarily in Western or Asian populations, this study provides region-specific MCHr validation in a Saudi Arabian cohort—an underrepresented group in anemia diagnostics research. By applying clinically relevant ferritin and TSAT thresholds, our stratified ROC analysis demonstrates MCHr's diagnostic strength, particularly in the context of inflammation. These findings extend the existing literature and support localized clinical implementation. Incorporating MCHr into anemia management protocols across CKD stages may enhance diagnostic precision, reduce dependence on inflammation-sensitive markers like ferritin, and facilitate more cost-effective, individualized use of intravenous iron and ESAs. Given its automation, low cost, and real-time reflection of marrow iron availability, MCHr is well suited for routine use in dialysis settings.

Furthermore, emerging data suggests its potential applicability in peritoneal dialysis and in patients with terminal allograft failure, although additional studies are needed to confirm its utility in these populations. Future interventional trials should investigate MCHr's role in long-term outcomes such as cardiovascular risk, ESA responsiveness, and mortality.

## 5 Conclusions

This study provides compelling evidence that the MCHr is a superior diagnostic marker for detecting iron-restricted erythropoiesis in patients undergoing hemodialysis. Compared to traditional biomarkers such as serum ferritin and TSAT, the MCHr demonstrated higher diagnostic accuracy, particularly in inflammatory states where conventional markers often fail to provide accurate results. Its real-time reflection of functional iron availability and independence from acute-phase reactants make MCHr a valuable addition to the anemia diagnostic toolkit in CKD care.

The optimal diagnostic cut-offs identified in this study, particularly < 31.2 pg for absolute iron deficiency and < 23.95 pg for functional deficiency when TSAT < 20%, demonstrated excellent sensitivity and specificity. These results are consistent with previous findings and confirm MCHr's reproducibility across diverse populations and clinical contexts. The robust correlation of MCHr with key hematologic and iron indices further underscores its clinical relevance.

Incorporating MCHr into routine anemia management for CKD patients offers the potential for more accurate diagnoses, timely intervention, and better monitoring of treatment responses. Future longitudinal studies should explore the dynamic role of MCHr in guiding therapeutic decisions, especially in conjunction with ESAs and intravenous iron therapy. Moreover, standardizing reference intervals across platforms and integrating inflammation-specific indicators will enhance MCHr's utility as part of a personalized approach to anemia care in chronic kidney disease.

## Data Availability

The original contributions presented in the study are included in the article/supplementary material, further inquiries can be directed to the corresponding authors.
